# Mixed phenotype acute leukemia with PML-RARα positive: a case report and literature review

**DOI:** 10.1186/s13039-021-00530-9

**Published:** 2021-02-11

**Authors:** Xiaolong Zheng, Huafei Shen, Mingyu Zhu, Yuanfei Shi, Huanping Wang, Zhimei Chen, Xin Huang, Yungui Wang, Jie Jin, Wanzhuo Xie

**Affiliations:** Department of Hematology, The First Affiliated Hospital, Zhejiang University School of Medicine, #79 Qingchun Road, Hangzhou, 310003 Zhejiang Province People’s Republic of China

**Keywords:** Acute leukemia, T/myeloid subtype, Mixed phenotype acute leukemia, PML-RARα, t(15;17), Acute biphenotypic leukemia, Case report

## Abstract

Mixed phenotype acute leukemia (MPAL) is an uncommon type of leukemia. It is one kind of malignant clonal diseases that expresses more than one genealogical specific antigen simultaneously. Most MPAL patients are associated with clonal chromosomal abnormalities and molecular genetic changes, such as t(9;22) (q34;q11) and KMT2A (MLL) rearrangement. These specific abnormalities usually have important guiding significance in MPAL diagnosis, targeted therapy and prognosis judgment. In this paper, we reported a case of MPAL, T/myeloid (M5) with an unfrequent combination of PML-RARα positivity and t(15;17). The treatment was successful with chemotherapy for both AML and ALL with daunorubicin, cytarabine (DA) and vincristine, prednisone (VP). We reported here this suggestive MPAL case of rare disease condition and effective treatment, in order to provide experience for the early diagnosis and treatment of similar patients.

## Introduction

With the development of cytogenetics and molecular biology, researchers have gradually strengthened their understanding of acute leukemia (AL). Morphology, immunology, cytogenetics, and molecular biology (MICM) are widely used in the world, which is not only of great significance to study the pathogenesis and biological characteristics of leukemia, but also of practical value to guide clinical treatment and prognosis judgment. According to its basic immunophenotype, AL is usually classified as acute myeloid leukemia (AML), acute B lymphoid leukemia (B-ALL), and acute T lymphoid leukemia (T-ALL). However, there is a type of ambiguous lineage, which we call mixed phenotype acute leukemia (MPAL). MPAL is an extremely rare type, accounting for about 2–5% of all AL [1, 2]. It is characterized by the detection of at least two of three expression markers in myeloid lineage, B lineage and T lineage.

The current diagnosis of MPAL is mostly based on the relevant standards revised by World Health Organization (WHO) in 2016. Based on the updated WHO classification of hematological malignancies, MPAL can be divided into several subtypes including MPAL with t(9;22)(q34.1;q11.2); BCR-ABL1, MPAL with t(v;11q23.3); KMT2A rearranged, MPAL, B/myeloid, not otherwise specified (NOS) and MPAL, T/myeloid, NOS [3]. MPAL is one of highly heterogeneous malignancies, and the clonal origin of MPAL cells is still unclear. It may be derived from early hematopoietic stem cells and differentiate into myeloid and lymphoid leukemia cells during the development of AL. There is at present no unified treatment for this special type of leukemia. It is controversial on whether MPAL should be treated with a single chemotherapy or combined with chemotherapy for both lymphoid and myeloid leukemia, and whether bone marrow or peripheral blood stem cell transplantation is required. Thankfully, cell and molecular genetic abnormalities such as chromosomal translocations and gene mutations can be detected in most MPAL patients, which are of great significance for guiding the treatment and prognosis of MPAL patients [2, 4].

Here we reported an extremely rare case of MPAL, T/AML(M5) with PML-RARα rearrangement and t(15;17). We discussed his diagnosis, treatment process and outcome in detail, and combined with literature review, in order to provide experience for the early diagnosis and treatment of similar patients.

## Case report

A 35-year-old man was admitted to the Department of Hematology, The First Affiliated Hospital, College of Medicine, Zhejiang University, in May 2020, with bleeding gums of 3-day duration. Physical examination showed scattered petechiae throughout the body. Routine laboratory tests revealed severe thrombocytopenia (platelets 8*10^9^/L), abnormal white blood cell counts (6.43*10^9^/L; 12.3% lymphocytes, 5.21% monocytes) and mild anemia (hemoglobin 100 g/L). Coagulation studies showed the international standardized ratio (INR) increased (1.18) and fibrinogen decreased (1.58 g/L, normal range: 2.00–4.00 g/L), the others including thrombin time (TT), prothrombin time (PT) and activated partial thromboplastin time (APTT) were normal. D-Dimer was raised to 11,200 μg/L FEU (normal range: 0–700 μg/L FEU). Lactate dehydrogenase (LDH) was slightly up to 385U/L (normal range: 120–250 U/L).

### Morphology

**Fig. 1:**
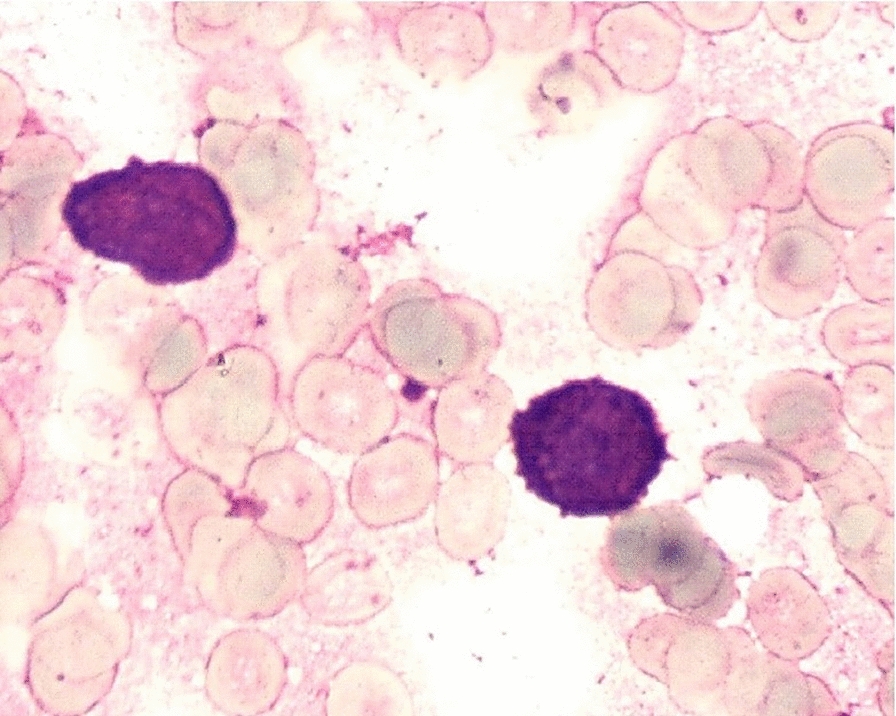
Bone marrow aspiration revealed morphological findings compatible with AML-M5b

A bone marrow aspirate showed monocyte proliferation was significantly active, with 72% of primitive monocytes + immature monocytes and 14% of mature monocytes, while other lines was inhibited. Moreover, Cytochemical staining suggested that myeloperoxidase (MPO), sudan black B stain (SB), nonspecific esterase (NSE) and NaF inhibit test were all positive. Based on bone marrow appearance, it was considered as acute nonlymphoblastic leukemia, morphologically resembling AML-M5b (Fig. 1).

### Immunology

**Fig. 2:**
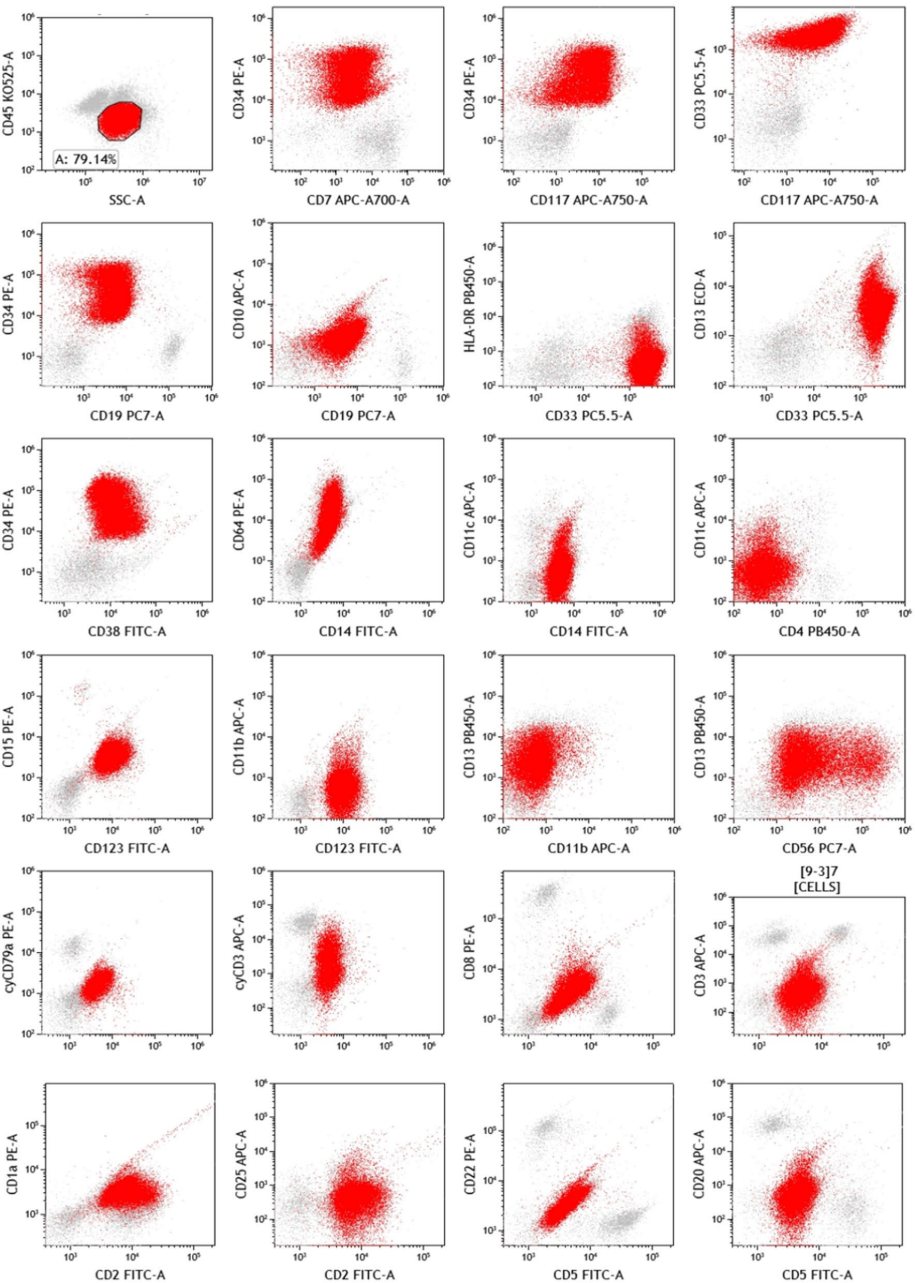
Immunophenotype with flow cytometry analysis suggested the possibility of mixed T/ myeloid leukemia

Immunophenotype with flow cytometry analysis found the protocell population accounted for about 79% of non-erythroid cells, expressing CD117, CD34, CD33, CD13, CD19 (weak), CD7, CD123, CD14, CD4, CD2, CD1a, MPO, cyCD3, and CD56 (sectional), which suggested the possibility of mixed T/ myeloid leukemia (Fig. 2).

### Cytogenetics and molecular biology

**Fig. 3:**
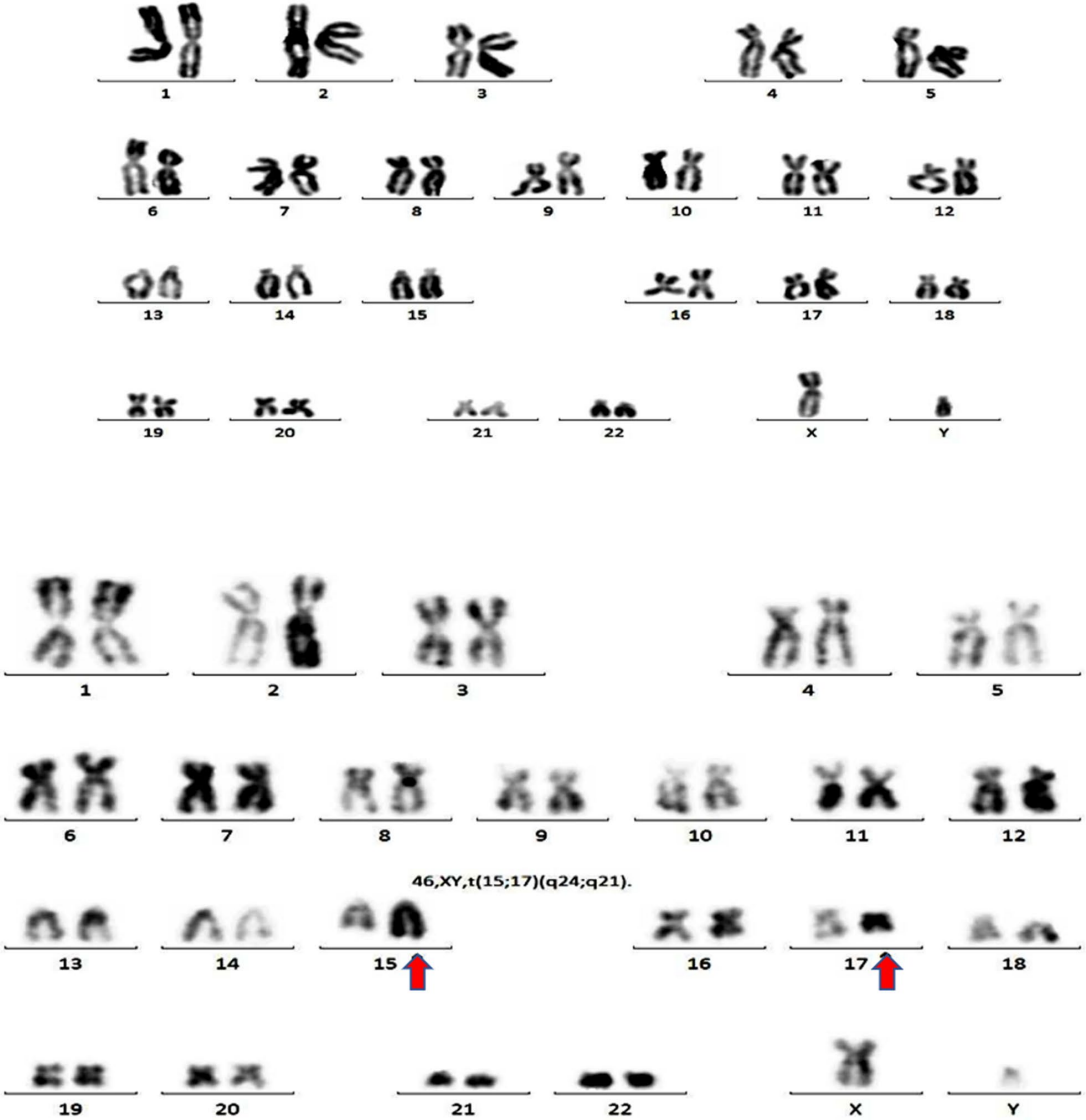
The karyotype is shown as 46,XY,t(15;17)(q24;q21)[3]/46,XY[9]. The retinoic acid receptor-α gene (RARα) on chromosome 17 is translocated transversely with the promyelocytic leukemia gene (PML) on chromosome 15

Chromosomal analysis with G-banded karyotype of bone marrow cells showed 46, XY, t(15;17)(q24;q21)[9]/46, XY[9] (Fig. 3). Fluorescence in situ hybridization (FISH) confirmed that PML/RARα was positive (note: the quality of FISH image was not optimal, however). The positive rate of PML/RARα short type was 39%, while that of long type was negative. There was evidence of FLT3-ITD mutation and no evidence of IDH1, IDH2, CEBPA, c-kit or NPM1 mutations.

### Clinical course

Based on his various auxiliary examinations, the diagnosis of MPAL was established. Then he was treated with daunorubicin, cytarabine (DA) and vincristine, prednisone (VP)(daunorubicin 120 mg Day 1–3 + cytarabine 100 mg Day 1–5; vincristine 4 mg Day 1, 8, 15 + prednisone 10 mg Day 1–21). The corresponding symptomatic support treatment also benefited the patient. The patient was subsequently treated with all-trans retinoic acid (ATRA) for maintenance therapy. There was no fever or bleeding during the whole treatment. After one course of chemotherapy, the patient's complete blood count (CBC) returned to be normal. Bone marrow examination revealed a primordial granulocyte ratio of less than 5%. Thanks to the timely diagnosis of the disease and effective treatment regimens, the patient finally achieved complete remission (CR). At present, the patient has remained first CR during the over 3 months.

## Discussion

MPAL has no specific chromosomal abnormalities. Owaidah et al. demonstrate that 68% of MPAL patients have clonal abnormalities, among which KMT2A translocation is the most common, followed by BCR-ABL [5, 6]. KMT2A rearrangement is more frequent in pediatric MPAL (especially infants), while BCR-ABL is more frequent in adults [7]. The case described herein is interesting because PML-RARα rearrangement complicated with t(15;17) in MPAL T/M cases is extremely uncommon. In general, PML-RARα fusion and t(15;17) are regarded as highly specific for acute promyelocytic leukemia (APL). There is few cases of AML with PML-RARα fusion and t(15;17) that were neither immunophenotypically nor morphologically consistent with APL [8]. To sum up, MPAL T/myeloid (M5) with PML-RARα positivity and t(15;17) is indeed quite rare.

The immunophenotype supported the diagnosis of MPAL T/M. In addition, we concerned that CD7 and CD34 were highly expressed in this case. CD7 is considered to be one of the T lymphoid-associated antigens, but it is not as specific as CD3. AML with CD7 high expression is generally connected with poor prognosis [9, 10]. Expression of CD34 also predicted unsatisfactory outcome. Furthermore, some researchers demonstrated that the positive rate of CD34 in MPAL was as high as 84% [11–14]. However, individual case report cannot verify these conclusions. The stratification of MPAL risk level needs more cases series and analytical studies to develop.

FLT3 is one of the most common mutations in AML, whose occurrence is often associated with poor prognosis [15, 16]. However, due to the rarity of MPAL, there are few studies on FLT3 mutation in MPAL. Zhang et al. indicated that ITD mutation was the dominant FLT3 mutation in MPAL patients [17]. Mutation in FLT3-ITD was also detected in this case. Some researchers believed that stem cell transplantation was more beneficial than chemotherapy for AML patients with FLT3-ITD mutation [18]. However, whether that applies to MPAL patients is worth further study.

MPAL is characterized by unique clinical and biological characteristics, with higher incidence in adults than in children, which is generally associated with worse prognosis [4]. There may be several reasons for the poor prognosis. First, the leukemia stem cells of MPAL are primitive pluripotent progenitors, which replicate too slowly to be resistant to chemotherapy. Second, due to the transformable phenotype, MPAL cells are capable to adapt to therapy. Third, a part of MPAL can highly express resistance-conferring P-glycoprotein [19, 12]. Therefore, the choice of chemotherapy regimen has always been a major difficulty in MPAL treatment. Gerr H, Rubnitz JE and their coworkers indicated that ALL-directed chemotherapy usually showed better outcome than AML-directed therapies in children. If the initial chemotherapy regimen was not effective, the conversion regimen could be chosen (from ALL-directed switch to AML-directed or vice versa). More than half of patients were able to achieve CR in the second regimen [20, 21]. Nevertheless, some researchers revealed that combined AML/ALL type regimens were more effective than single regimen for adult patients. The CR rate of combined chemotherapy was the highest (71%), followed by ALL-directed (64%), and the lowest was AML-directed (33%) [14]. Moreover, Zhang and his coworkers demonstrated that combined-type regimens or ALL-based protocols are effective for the treatment of adult MPAL [14] . As such, we managed the patient with combined chemotherapy for ALL and AML, which contributed to his achievement of CR.

In conclusion, we reported a pretty unfrequent case of MPAL T/M with PML/RARα rearrangement and t(15;17). The outcome of this patient was markedly satisfactory with combined AML/ALL type regimens (DA + VP) and ATRA as well.

## Data Availability

Not applicable.
